# The quest for effective Ebola treatment: Ebola VP35 is an evidence-based target for dsRNA drugs

**DOI:** 10.1038/emi.2014.77

**Published:** 2014-10-29

**Authors:** William M Mitchell, William A Carter

**Affiliations:** 1 Department of Pathology, Microbiology and Immunology, Vanderbilt University, Nashville, TN 37232, USA; 2 Hemispherx Biopharma, Inc., Philadelphia, PA 19103, USA

**Dear Editor,**

For decades, Ebola members (EBOV) of the *Filoviridae* family of highly pathogenic, filamentous single-stranded RNA viruses have been responsible for sporadic self-limiting clusters of Ebola virus disease (EVD) in Central Africa. The current outbreak of EVD in West Africa has risen to epidemic status and is considered by the World Health Organization to be a public health emergency of international concern. This level of concern by the World Health Organization and other health-care authorities and providers is based on the high lethality of EBOV-Zaire in humans (up to 90%), its high communicability though body fluids, the high incidence of infection in primary health-care workers and the total lack of proven efficacious drugs or vaccines. Although there are a variety of potential EBOV inhibitors in the drug development pipeline, none have sufficient human safety data, which complicates estimates of their efficacy in the current crisis situation in West Africa.^1^ One such experimental drug is the Zmapp monoclonal antibody cocktail (*n*=3), which was shown to be highly effective in reversing advanced EVD in non-human primates.^2^ However, the rapid acquisition of EBOV sequence variation recently demonstrated during genomic surveillance of the current EVD epidemic suggests that drug development dependent on viral non-variant protein sequences may be problematic.^3^ In this Letter, we briefly address the evidence-based potential of several Toll-like receptor 3 agonists in late-stage clinical development for reducing the morbidity and mortality of EVD.

Death from EBOV infection is associated with markedly impaired coagulation and innate immunity cascades, increased production of pro-inflammatory cytokines, profound immune suppression resulting in peripheral T lymphocyte apoptosis and a lack of adaptive immunity.^4^ By contrast, survivors of infection by EBOV develop an effective immune response with the production of EBOV neutralizing antibodies. Early events in EBOV infection influence the patient's ability to develop an effective immune response. The success of EBOV replication is dependent on viral inhibition of initial innate immune responses to infection. Disarming innate immune responses is a common mechanism employed by highly pathogenic human viruses, including those of the influenza and coronavirus families.^5^ EBOV is one of the more successful of the emerging highly pathogenic viruses in evasion of innate immune interference. Viral protein (VP35), an essential component of the EBOV replication matrix, represents an evidence-based target for the potential reduction of the morbidity and mortality of EVD.

Double-stranded RNA (dsRNA) is a common component of viral replication, which initiates systemic signaling cascades that normally activate interferon (IFN) regulatory factors leading to the production of IFN-α/β (type I IFNs). The IFNs activate multiple IFN response pathways necessary to inhibit viral replication, including transitory expression of enzymes such as 2′–5′ adenylate synthetase and protein kinase R, which require dsRNA as a cofactor for activity. To circumvent viral recognition by human cells, highly virulent viruses (including Ebola) have evolved different strategies to block the biological activities associated with the induction of IFN-α/β and the subsequent multiplicity of anti-viral responses and the initiation of adaptive immunity. VP35 is a multifunctional major virulence protein that is indispensable for EBOV replication as a component of the viral polymerase complex.^4^ This factor also counteracts the host innate immune response by blocking the cellular production of and responses to type I IFN transient gene activation. Key components of the innate immune response to viral infection include activation of Toll-like receptor 3 and the helicases melanoma differentiation-associated protein 5 and retinoic acid-inducible gene1 by viral dsRNA.^5^ However, viral inhibition of these dsRNA responsive elements effectively disarms essential components of the innate immune response. In particular, a positively charged C-terminal amino acid motif (Figure 1) of VP35 binds to sequence-independent dsRNA, resulting in the suppression of multiple steps in the IFN signaling cascade, which otherwise would lead to a broad antiviral state with strong activation of both the innate and adaptive arms of the immune response.^8^ Thus, VP35 provides an attractive evidence-based target for antiviral interruption of EBOV, potentially ameliorating the pathogenesis of EVD.

Various forms of dsRNA have been studied as inducers of type I IFNs. Although the original dsRNA studied for efficacy in humans, Poly I:Poly C, was found to induce serious adverse events in humans that limited pharmaceutical development, several derivatives have survived the rigors of animal and clinical testing. Poly ICLC (Hiltonol^®^) (Oncovir, Inc., Washington, DC, USA) consists of Poly I:Poly C with non-covalent adduct polymeric chains of poly-*L*-lysine and carboxymethylcellulose. These adducts increase the drug half-life by steric hindrance of the phosphodiester backbone from RNAse hydrolysis. Hiltonol^®^ has been studied in clinical trials as a cancer therapy and as an adjuvant for cancer vaccines.^9^ Poly I:Poly C_12_U (rintatolimod, Ampligen^®^) (Hemispherx Biopharma, Inc., Philadelphia, PA, USA) was designed as an IFN inducer with a markedly reduced incidence of adverse advents compared to the parent compound Poly I:Poly C^8^ and is devoid of adducts. Both Hiltonol^®^ and Ampligen^®^ have demonstrated antiviral activity against a wide variety of DNA and RNA viruses in preclinical testing.

In animal studies, rintatolimd was efficacious as an epitope-expanding adjuvant for highly pathogenic avian influenza virus vaccines,^10^ and this drug is now being evaluated as an adjuvant in a variety of cancer vaccine trials. Rintatolimod has demonstrated activity in two double-blind, placebo-controlled clinical trials of chronic fatigue syndrome^8^ and is currently in an advanced clinical trial as a treatment for this condition. Hiltonol^®^ and/or rintatolimod experimental pharmaceuticals are attractive evidence-based candidates for the treatment of EVD on the expanded basis recently approved by the World Health Organization. The use of experimental pharmaceuticals beyond phase I in Food and Drug Administration-sanctioned clinical trials helps ensure the availability of products with established safety profiles sufficient for open-label clinical testing and analysis of efficacy. The use of potential drugs in clinical trials also helps ensure a reliable supply of clinical-grade drugs, which removes the ethical dilemma of patient selection for treatment.^1^

## Figures and Tables

**Figure 1:**
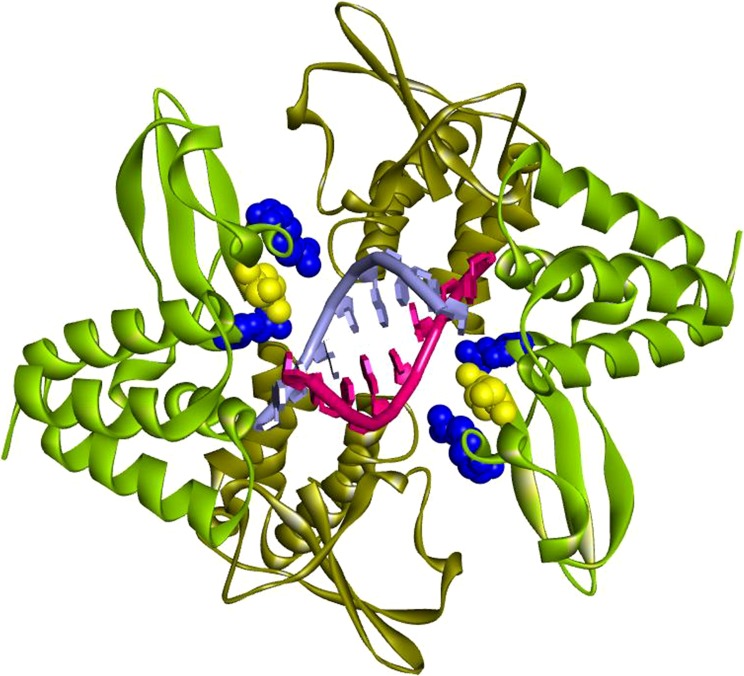
Structure of dsRNA C-terminal binding domain of EBOV VP35. Binding to dsRNA and IFN inhibition is dependent on a VP35 complex in which two VP35 molecules are paired in binding to a single RNA strand. Illustrated are the crystallographic coordinates of a truncated VP35 (residues 215–340) complex with an 8-bp dsRNA. Each dsRNA strand is depicted as red or blue-grey with similarly colored bases to indicate that binding of VP35 is nucleotide sequence independent. Three positively charged residues of VP35 are critical for binding to the phosphodiester dsRNA backbone, which results in inhibition of the initiation of innate immune responses including IFN induction. Arginines 312 and 339 on each binding homodimer (mint green) are represented as blue CPK van der Waals radii, with lysine 319 shown as a yellow CPK van der Waals radius. The second monomer (brown) of each pair does not make direct contact with dsRNA, although hydrogen bonds exist between the homodimers. Helical structures are represented as coils (α-helices) or flat bands (β-sheets). The VP35 binding affinity for dsRNA is size-dependent (2.8 nM for 500-bp dsRNA versus 3.2 nM for 50-bp dsRNA).^6^ The illustration is based on PDB ID: 3L25 coordinates^7^ using Accelrys version 3.5 software. CPK, Corey–Pauling–Koltun.
